# Nudging clinical supervisors to provide better in-training assessment reports

**DOI:** 10.1007/s40037-019-00554-3

**Published:** 2019-12-17

**Authors:** Valérie Dory, Beth-Ann Cummings, Mélanie Mondou, Meredith Young

**Affiliations:** 1 Department of Medicine and Centre for Medical Education; Faculty of Medicine, McGill University, Montreal, QC Canada; 2 Undergraduate Medical Education, Department of Medicine, and Institute of Health Sciences Education; Faculty of Medicine, McGill University, Montreal, QC Canada; 3 Department of Medicine and Institute of Health Sciences Education; Faculty of Medicine, McGill University, Montreal, QC Canada

**Keywords:** Workplace-based assessment, Faculty development, Feedback

## Abstract

**Introduction:**

In-training assessment reports (ITARs) summarize assessment during a clinical placement to inform decision-making and provide formal feedback to learners. Faculty development is an effective but resource-intensive means of improving the quality of completed ITARs. We examined whether the quality of completed ITARs could be improved by ‘nudges’ from the format of ITAR forms.

**Methods:**

Our first intervention consisted of placing the section for narrative comments at the beginning of the form, and using prompts for recommendations (Do more, Keep doing, Do less, Stop doing). In a second intervention, we provided a hyperlink to a detailed assessment rubric and shortened the checklist section. We analyzed a sample of 360 de-identified completed ITARs from six disciplines across the three academic years where the different versions of the ITAR were used. Two raters independently scored the ITARs using the Completed Clinical Evaluation Report Rating (CCERR) scale. We tested for differences between versions of the ITAR forms using a one-way ANOVA for the total CCERR score, and MANOVA for the nine CCERR item scores.

**Results:**

Changes to the form structure (nudges) improved the quality of information generated as measured by the CCERR instrument, from a total score of 18.0/45 (SD 2.6) to 18.9/45 (SD 3.1) and 18.8/45 (SD 2.6), *p* = 0.04. Specifically, comments were more balanced, more detailed, and more actionable compared with the original ITAR.

**Discussion:**

Nudge interventions, which are inexpensive and feasible, should be included in multipronged approaches to improve the quality of assessment reports.

## Background

Competency-based medical education relies heavily on workplace-based assessment to guide learning and inform decisions about learners’ attainment of competence [1]. Workplace-based assessment is traditionally documented at the end of a clinical rotation in an in-training assessment report (ITAR—previously referred to as in-training evaluation report or ITER)[2]. Although the shift to competency-based medical education is leading to more frequent documentation of specific assessment events, ITARs continue to play a role in synthesizing assessments for decision-making and for providing formal feedback to learners [2].

To effectively support decision-making and feedback, ITARs must meet quality standards [3]. During a reform of our undergraduate medical education program at McGill University, the committee responsible for curriculum renewal in clerkship (i.e. the clinical phase of the curriculum, in the third and fourth years of a 4-year curriculum) identified several issues with our ITARs. Specifically, numeric grades appeared inflated (with average ratings in the ‘exceeds expectations’ range), and comments were considered generic and uninformative by both course directors and student representatives.

Faculty development has been championed as a means of ensuring quality of workplace-based assessment in general [4] and of ITAR completion in particular [5]. However, organizing effective faculty development initiatives that reach the large numbers of clinical supervisors completing ITARs is resource-intensive. Nudge theory proposes that small, low-cost changes to the **‘environment’** in which decisions are made can increase the likelihood that individuals will behave in desired ways without coercion [6]. Examples of nudges include presumed consent for organ donation with means for individuals to register refusal (i.e. using default options to influence behaviour), providing the estimated number of calories burned on gym equipment (i.e. providing feedback to influence behaviour), or stating the rate of tax compliance in a letter to tax-payers (i.e. using social norms to influence behaviour)[6]. Nudges have proved effective in influencing a variety of behaviours in diverse domains from nutrition (e.g. providing smaller plate sizes or portions, modifying food labelling) [7] to the environment (e.g. providing social comparisons in electricity bills, making the default energy provider an environmentally responsible one) [8]. This project examined whether nudges, i.e. changes to the environment in which clinical supervisors provide assessment data, specifically changes to the structure of ITAR forms used for undergraduate clinical placements, could improve the quality of the data generated from ITARs.

## Development and implementation of the nudge interventions

### *Nudge interventions (*Tab. 1*)*

**Table 1:** Overview of the format of in-training assessment forms used

	Baseline	Intervention 1	Intervention 2
Overall scoring	5‑point scale	Pass-fail	
Comment boxes	At the end	At the beginning	
Number of comment boxes	1	4	2
Number of specific checklist items	8	27	7
Number of specific rating scale items (number of points on scale)	12 (5)	0	0

As part of our curriculum reform, the program shifted to pass-fail grading in an effort to encourage students to prioritize learning over competition for grades [9]. This implied concurrent changes to the ITARs, aimed at encouraging supervisors to provide more narrative comments, more balanced comments (i.e. including both strengths and areas for improvement), and more actionable comments (i.e. with specific recommendations to learners about *how* to improve, not just *what* to improve), which learners could use to direct their learning.

Our original locally developed ITAR form had 8 checklist items and 12 5-point rating scales items, followed by a single free-text comment box, and an overall rating item. In 2015 (Intervention 1), we moved the comments section to the beginning of the form to ‘nudge’ supervisors to provide more narrative comments. We also split the single comment box into four distinct comment boxes (Keep doing, Do more, Do less, Stop doing), to ‘nudge’ supervisors to write more balanced and actionable comments. Specific items (*n* = 27) were all in checklist format (attained course objectives/has not yet attained course objectives/not observed) to reflect the program’s shift to pass-fail grading.

In 2016 (Intervention 2), we addressed feedback (provided by clinical supervisors to course directors) about the comment boxes being cumbersome, by replacing our previous four free-text boxes with two boxes: one to describe performance and one to provide recommendations (which was subdivided in two boxes: Keep doing or do more, Do less or stop doing). To support assessors in developing narrative comments, we provided a hyperlink to a detailed assessment rubric. To further increase the relative emphasis on narrative comments, we shortened the checklist from 27 to 7 items (one for each CanMEDS role [10]).

### Evaluation plan

Our medical students (class size of around 190) rotate through six clinical placements in the first year of clerkship, generating approximately 1140 forms for these clinical rotations every academic year. In order to compare the quality of data generated from the three different assessment forms (baseline; intervention 1, intervention 2), we randomly sampled 120 forms from three successive academic years, for a total of 360 forms. To ensure that the sample was representative in terms of the different clinical specialties and the time of year (student performance should increase and assessors may become more familiar with the forms as the year progresses), we stratified our sample by course (*n* = 6) and trimester (*n* = 3). The assessment administrator extracted the forms for each clinical course from the learning management system, split them by trimester, and randomly selected a sample of 6–7 forms for each trimester, i.e. selecting 20 forms for each clinical course, for three academic years. She then de-identified the forms.

Two raters (the first author and an academic associate from our Assessment and Evaluation Unit) independently rated the quality of each completed form, using Dudek et al.’s Completed Clinical Evaluation Report Rating (CCERR) rating scale [11]. The CCERR rating scale includes 9 items, each rated on a 5-point scale, with 1 anchored as ‘not at all’, 3 ‘acceptable’, and 5 ‘exemplary’. The total score is the sum of all items and can range from 9 to 45. The CCERR items were developed using a focus group and modified Delphi technique with members of three stakeholder groups (i.e. educational leaders, clinical supervisors, and residents) [11]. An initial study found that it could reliably discriminate between forms and that raters were consistent in its use [11]. Dudek et al. also provided convergent validity evidence as CCERR scores correlated well with expert judgements of form quality [11]. The CCERR scale has subsequently been used to describe ITAR quality in different settings and to determine the effect of interventions on ITAR quality [5, 12–14].

We examined our inter-rater agreement using a two-way mixed effects model (random rater effects and fixed rating instrument effects) intraclass correlation coefficient, and found high levels of inter-rater agreement (ICC for average measures = 0.96). We used the average of both raters’ scores for subsequent analyses. We tested for differences between the three versions of the ITAR forms using a one-way ANOVA for the total CCERR score, and MANOVA for the nine CCERR item scores.

## Evaluation findings

**Fig. 1:**
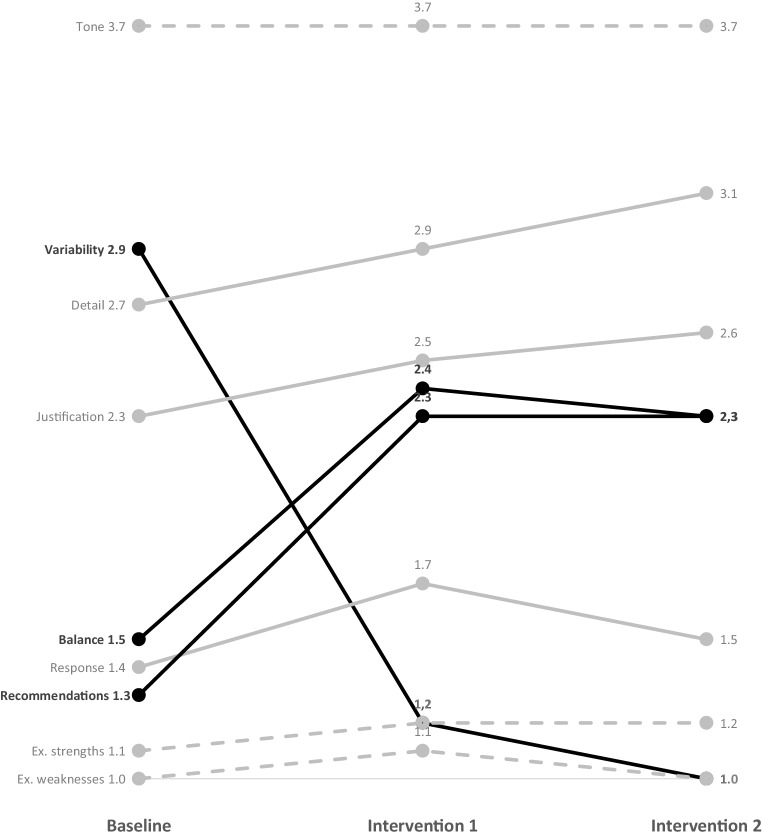
Average scores on the nine items of the Completed Clinical Evaluation Report Rating (CCERR) scale [11]. *Dash‐lines* indicate non‐significant results, *continuous lines* statistically significant results, with *black lines* indicating steeper changes

The overall quality of our sample of completed ITARs was low, with total scores in the 18–19 range out of a maximum score of 45 (details provided in Tab. 2 and Fig. 1). Changes in forms successfully increased the balanced (from 1.5 (SD 0.7) to 2.4 (SD 0.8) and 2.3 (SD 0.7), *p* < 0.001) and actionable nature of comments (from 1.3 (SD 0.6) to 2.3 (SD 0.8) and 2.3 (SD 0.6), *p* < 0.001), and slightly increased the overall level of detail provided (from 2.7 (SD 0.7) to 2.9 (SD 0.7) and 3.1 (SD 0.6), *p* < 0.001). Scores on one item (‘Checklist/numeric ratings show sufficient variability to allow identification of relative strengths and weaknesses of the trainee’) decreased (from 2.9 (SD 0.9) to 1.2 (SD 0.4) and 1 (SD 0.2), *p* < 0.001). This was inevitable with the change from 5‑point rating scales to a checklist format (a change which was intended to reflect the pass-fail philosophy of the curriculum). The overall quality of completed ITARs increased (from 18.0 (SD 2.6) to 18.9 (SD 3.1) and 18.8 (SD 2.6), *p* < 0.05).

**Table 2:** Comparison of the quality of completed in-training assessment reports across the three versions of the forms

Completed Clinical Evaluation Report Rating (CCERR) rating scale [11]	Maximum possible score	Baseline	Intervention 1	Intervention 2	F‑value MANOVA	*p*-value MANOVA
		Average score (standard deviation)				
Checklist/numeric ratings show sufficient variability to allow identification of relative strengths and weaknesses of the trainee	** 5**	** 2.9** **(0.9)**	** 1.2** **(0.4)**	** 1** **(0.2)**	**357.59**	**<0.001**
Comments are balanced providing both strengths and areas for improvement	** 5**	** 1.5** **(0.7)**	** 2.4** **(0.8)**	** 2.3** **(0.7)**	**47.61**	**<0.001**
The trainee’s response to feedback and/or remediation during the rotation is described in the comments	** 5**	** 1.4** **(0.8)**	** 1.7** **(0.9)**	** 1.5** **(0.8)**	**3.47**	**0.03**
Comments justify the ratings provided	** 5**	** 2.3** **(0.5)**	** 2.5** **(0.5)**	** 2.6** **(0.4)**	**6.97**	**<0.001**
Clearly explained examples of strengths using specific descriptions (not generalizations) are provided in the comments	5	1.1 (0.5)	1.2 (0.5)	1.2 (0.5)	1.45	0.24
Clearly explained examples of weaknesses using specific descriptions (not generalizations) are provided in the comments	5	1 (0.2)	1.1 (0.3)	1 (0.2)	1.62	0.20
Concrete recommendations for the trainee to attain a higher level of performance are provided	** 5**	** 1.3** **(0.6)**	** 2.3** **(0.8)**	** 2.3** **(0.6)**	**79.09**	**<0.001**
Comments are provided in a supportive manner	5	3.7 (0.3)	3.7 (0.4)	3.7 (0.3)	1.75	0.18
Overall, this ITAR provides enough detail for an independent reviewer to clearly understand the trainee’s performance on the rotation	** 5**	** 2.7** **(0.7)**	** 2.9** **(0.7)**	** 3.1** **(0.6)**	**10.35**	**<0.001**
					F‑value ANOVA	*p*-value ANOVA
Total score	**45**	**18.0** **(2.6)**	**18.9** **(3.1)**	**18.8** **(2.6)**	**3.33**	**0.04**

Intervention 1 included splitting the comment box into four specific boxes and moving them to the beginning of the form, and replacing rating scale items to checklist items (pass-fail grading). Intervention 2 included simplifying the comment boxes, reducing the number of checklist items, and providing a hyperlink to a detailed assessment rubric

Statistically significant results are in bold

## Reflection

Changes to the ITAR forms improved the quality of reports. The improvements were only detectable following the first intervention, suggesting that the location of the comment boxes and having at least one box specifically for recommendations were the effective components of our interventions. Reducing the length of the checklist had little impact as did the provision of a hyperlink to a detailed rubric describing different levels of student performance. Although we found an effect of our nudge interventions overall, the magnitude of the effect was small. This could be due in part to contextual factors. Many of our students apply to residency programs that request copies of applicants’ ITARs on top of their academic transcripts. This increases the stakes of ITARs and could discourage supervisors from documenting feedback targeting areas for improvement, despite nudges to do so in ITAR forms. The magnitude of the effect should also be considered in light of findings from other studies using the same instrument to measure ITAR quality. As a point of comparison, Dudek et al. [5] reported an increase in the quality of completed ITARs using the CCERR instrument from 18.90 to 21.74 following a faculty development workshop. This increase was threefold larger than the size of increase we found by changing the forms, but the costs incurred were likely also much larger, and the number of faculty members participating in the workshop was likely much lower than our faculty-wide nudge experiment.

One limitation to our findings is the reliance on a naturalistic experiment: we were unable to deploy different forms for the same cohort of students. The interventions occurred at the same time as other curricular changes, which may have also influenced our findings. Clinical supervisors may have been particularly stretched with the implementation of revised curricular outcomes and new teaching activities to engage fully with the revised assessment forms. If anything, this would have reduced the impact of our nudges. The concurrent shift to pass-fail may also have had an impact. It is conceivable that shifting to pass-fail would increase the perceived importance and stakes of narrative comments, as comments would become the only discriminating information about students’ clinical performance available in students’ applications to residency programs. On the one hand, this could have motivated clinical supervisors to provide more detailed comments, regardless of changes to the forms. On the other it could have led them to avoid providing negative feedback. In fact, we found that the balance of comments improved more than the level of detail, suggesting the shift to pass-fail had minimal impact. Finally, we could not blind those who rated the completed forms to the form structure.

Nonetheless, this study suggests that nudges such as form changes can be used to improve the quality of information provided to learners and decision-makers. The effects we found were small. However, no single intervention, including faculty development and feedback, has so far led to the substantial increases in the quality of ITAR completion that would be desirable [5, 12, 14]. ITARs play an important role both as assessment-*for*-learning and assessment-*of*-learning. The provision of performance information as well as specific recommendations should be helpful to learners, although we acknowledge that providing information alone is insufficient to change behaviour [15, 16]. ITARs are also an important data source for progress decisions [2, 17] and narrative comments in particular can be effectively interpreted by experienced clinical supervisors [18–20]. In light of the importance of the information collected in ITARs and its widespread inadequacies, we suggest that multipronged interventions—including feasible and low-cost nudges alongside more resource-intensive faculty development—should be deliberately implemented. Other interventions aligned with nudge theory should also be considered. Providing assessors with feedback has already been proven effective in improving the quality of ITARs [12], and providing assessors with information about the performance of their peers, i.e. using social norms [6], could also be explored. Although each intervention may have small effects in isolation, combining them could result in meaningfully improved feedback and better decision-making in health professions education.
